# Weight management in a cohort of Irish inpatients with serious mental illness (SMI) using a modular behavioural programme. A preliminary service evaluation

**DOI:** 10.1186/1471-244X-8-76

**Published:** 2008-09-15

**Authors:** Chris J Bushe, Dermot McNamara, Cliff Haley, Mary Fleming McCrossan, Pat Devitt

**Affiliations:** 1 Eli Lilly and Company Ltd, Adelaide Road, Dublin, Ireland; 2 Psychiatry Dept, Letterkenny General Hospital, Donegal, Ireland; 3 Nursing Dept, Cleary House, Letterkenny, Donegal, Ireland; 4 Psychiatry Dept, Adelaide and Meath Hospital, Tallaght, Dublin 24, Ireland

## Abstract

**Background:**

Weight gain is commonly observed during psychotropic treatments for chronic forms of severe mental illness and is most rapid during the early treatment phases. All formats of behavioural weight intervention programmes have suggested that weight gain can be prevented or reversed in some patients. There is no data on these programmes in acutely unwell inpatients whom may be the major beneficiaries.

**Methods:**

A modular behavioural intervention programme (Solutions for Wellness) used in SMI outpatients since 2002 in Ireland has been adapted for inpatient use. Preliminary data is reported from 5 centres in Ireland.

**Results:**

In 47 inpatients the mean weight change was +0.26 kg (SD 2.02) with a median change of 0 kg. Mean follow-up was 23.7 (SD 21.6) days, and median 14 days (range 6–98 days). There was no difference in mean weight change in those patients involved for > 35 days compared with < 35 days (+0.26 kg; 0.25 kg; p = 0.5). Weight loss or maintenance was seen in 70% of patients.

**Conclusion:**

These preliminary data are supportive of the concept that acutely unwell inpatients with SMI may engage with a behavioural weight programme. Weight change observed contrasts with the significant weight gain often seen in most subjects. Further clinical trials are warranted.

## Background

There has been increasing recognition that cardiovascular mortality is significantly increased in patients with severe mental illness (SMI) [1-3]. There are complex reasons for this that include genetic predisposition, smoking, poor dietary choices, decreased physical activity and weight gain observed with most psychotropic treatments [4]. The consensus view is that cardiovascular risk factors in SMI patients need to be reduced [2,5]. Weight gain prevention is one of the more easily defined and measured problem areas.

Patients with schizophrenia are generally of normal BMI at illness onset and males may even be underweight [6]. There is clear recognition that patients treated in their early phase of psychosis gain significant weight during the first year of treatment with the recent CAFÉ study reporting 80% olanzapine subjects, 50% quetiapine and 58% risperidone subjects gaining > 7% of their body weight in the first 12 months [7] in the absence of any preventative intervention. In addition although weight gain is maximal during the early months of initiating antipsychotic treatment, in a large 3 year naturalistic study an increasing number of subjects on all medications continued to gain weight during the 3 year study [8,9], and [10].

These efficacy based clinical trials both randomised and observational have to date not been able to encompass any preventative measures to reduce weight gain. Despite this a variety of weight and lifestyle management programmes have been developed worldwide in outpatients and broadly have produced good results [11-16]. The forms of intervention range from weekly group sessions using behavioural models through to one to one nursing sessions and telephone/internet based models [17].

In Ireland a lifestyle management programme *Solutions for Wellness *has been operating in outpatients with forms of severe mental illness (SMI) since 2002 and recently reported preliminary data in the outpatient subject cohort [15]. The programme has been slightly adapted to be used in an inpatient setting with the rationale of earlier intervention. We describe preliminary results from the initial cohort of patients evaluated. The premise being evaluated is that early weight gain may be prevented or reduced in some patients.

## Methods

A group programme (Acute Solutions for Wellness) designed to address weight and other cardiovascular risk factors commenced in 2005 in a number of geographically diverse centres in Ireland in inpatients with acute forms of severe mental illness (SMI). Five centres of those taking part in the programme agreed to provide their data for a preliminary service evaluation. Data was not routinely recorded in other centres. There was no restriction placed on the precise diagnoses to which the intervention would be offered to but it was expected that most patients would have schizophrenia or severe affective disorders and was offered to suitable inpatients referred by the ward nursing staff. There was neither restriction on drug and non-drug treatments nor age restrictions. As this was not a clinical study total discretion for entry to the programme remained with the ward nursing staff. They chose subjects whom they considered were potentially suitable for the programme based on mental state. The groups commenced with a cohorts of patients at each centre whom progressed through the programme sequentially as a group.

The programme consisted of 8 modules to complete over 4 weeks, each module of 30 minutes covering dietary choices, appetite, physical activity and nutrition. The materials were adapted to be simplified from the outpatient version of the same service and in addition modules were designed to be 30 instead of 60 minutes each. The programme otherwise was unchanged. Groups were led by trained healthcare professionals usually nurse. The nurses received full training in the operation of the modular programme and worked from identical source material workbooks consisting of 20 page instructor guides describing each module individually. Patients also received written materials in the form of a 22 page workbook similarly describing in appropriate terminology each module separately. The 8 modules covered the topics:

1. Healthy living

2. Physical activity

3. The food pyramid

4. Recommended food servings

5. Fat and salt in your diet

6. Healthy and unhealthy eating habits

7. High fibre diet

8. Controlling your hunger

The patients received either twice weekly modules each 30 minutes or a single hourly session weekly combining two modules. Subject diagnoses were recorded from the notes.

### Ethics

Ethical approval was not sought as this service provision of SFW was deemed to be part of good clinical practice and service evaluations may be considered exempt from ethical review. The only difference being that the service was provided in secondary care rather than in primary care. Informed consent was obtained from the patients to use their data in an anonymised manner for the purpose of a service evaluation.

### Statistical Analysis

Our analysis has been predominantly descriptive, aiming to present the raw data as a series of weight or BMI results over a period of time. Our descriptive statistics are supported with standard deviations, median and significance is reported where appropriate.

## Results

Since 2005 data is available from 47 patients (26 females, 21 males) from 5 centres. Diagnoses were Schizophrenia in 17/47, affective disorders 25/47, non-psychotic diagnoses (personality disorder, anxiety, alcohol misuse) 5/47. Baseline mean weight 78.4 kg (SD 15.2 kg) and median 77 kg (range 47–135 kg).

**Figure 1:**
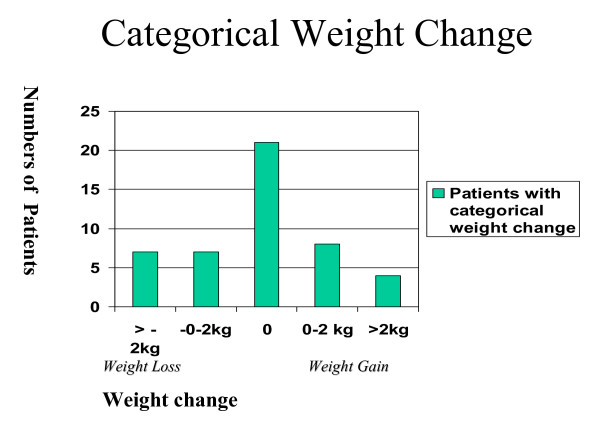
Length of engagement with programme by patients.

**Figure 2:**
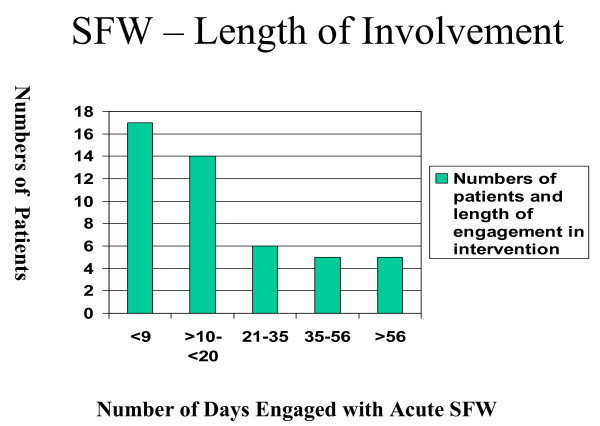
Categorical amounts of weight change in patients.

The mean weight change was +0.26 kg (SD 2.02) with a median change 0 kg. Paired Student's t-Test t = -0.624, df = 46, p = 0.950. Mean follow-up 23.7 (SD 21.6) days, median 14 days (range 6–98 days) with 16/47 engaging with the programme >=21 days (figure 1). The categorical weight changes at discontinuation with programme were weight gain in 14/47 (30%), weight maintenance in 21/47 (45%) and weight loss in 12/47 (25%). The categorical changes are shown in figure 2. The mean weight change in the 10/47 patients whom remained in the programme >=35 days was +0.25 kg, (SD 3.55) and in the 37/47 whom remained in the programme <=35 days +0.262 kg, (SD 1.43) (p = 0.50)

## Discussion

In this preliminary report an adapted lifestyle intervention programme has found promising results. At discontinuation of engagement with the programme only 14/47 (30%) patients had gained weight during a mean follow up of 24 days (median 14 days) and the remainder either maintained their weight or lost weight. The median weight change was 0 kg. Furthermore there were no differences between those patients who remained in the programme less than 35 days compared to the group whom engaged for greater than 35 days (p = 0.5). These data may hold relevance in that a major determinant of length of engagement with SFW was the time to discharge of the patients. At discharge there would be no certainty that continual engagement with any weight management of lifestyle intervention programme would be either offered or accepted.

There are few data which can be compared with our data as to our knowledge this is one of the first data sets that has examined a lifestyle intervention in an inpatient SMI cohort acutely unwell.

Some short term interventions have been successful in outpatients. In a 14-week open label study of patients switched to risperidone from olanzapine and randomised to receive a behavioural intervention, 27% of the active cohort lost > 5% body weight over 14 weeks [13] and in a 12-week intervention using a similar programme to Acute SFW in outpatients there was a mean weight loss of 2.7 kg [18]. The few studies that have examined interventions in early phases of illness are worth comparison. A study of 61 first-episode patients aged 16–45 years randomised to behavioural intervention or "usual care" over 12 weeks [19]. Only 40.7% of the intervention group gained > 7% of weight compared with 77% of the control group. Similar data was reported from a cohort of 50 patients randomised to intervention or usual care within 30 days of commencement on an antipsychotic and followed for 16 weeks [20]. 63% of the intervention cohort experienced no weight gain compared with 22% of the control patients (p = 0.009). In this study 70% of our cohort experienced no weight gain but only over mean 24 days follow up. A further encouraging finding was that the patients engaging with the programme for greater than 35 days had no greater weight gain than those engaging for less time. In a recent naturalistic study of an admixture of inpatients and outpatients (mean weight loss 2.64 +- 2.75 kg) BMI reduction over 12 weeks was greater in the outpatient cohort making these preliminary data even more encouraging and worthy of further research [21].

The literature on weight management programmes consists primarily of uncontrolled interventions, whose data seems promising and shorter term controlled trials but not conclusive of benefit. With a few reservations most data show encouraging results. A number of systematic reviews and reviews have consistently suggested some benefit from these programmes [14,22]. Recent controlled trials have also suggested more tangible gains from lifestyle programmes. Mauri et al, report a reduction in fasting insulin levels, insulin resistance (HOMA index) and improvement in insulin sensitivity but unchanged FPG in an olanzapine cohort randomised to a psycho educational programme for weight loss [23] for either 12 or 24 weeks. Menza found a reduction in glycosylated haemoglobin over 1 year using a multi-modal weight control programme (n = 31) from 5.35% to 5.11% (p = 0.001) [24].

The data from the UK programme Wellbeing support programme report 80% retention in the programme over 2 years and in addition to reduction of various cardiovascular risk factors; weight gain to any extent was measured in only 30.8 % [25]. Longer term compliance with these programmes may be crucial with recent data suggesting compliance is a significant factor [21,11], and [12]. An additional salient consideration is that Werneke et al in 2002 [26] and Faulkner in 2007 [27] concluded that there was not sufficient evidence to use any pharmacological agents for weight reduction or prevention.

In a Cochrane review in 2007 Faulkner found that only 5 trials out of 23 randomised interventions were behavioural in type and modest weight loss and prevention were reported [27]. The relevant caveats around these findings included small studies over short term periods with varying interventions, and longer term larger studies need to be undertaken before making definitive statements of benefit.

### Limitations

There are however significant limitations in our data that will need to be resolved in future controlled clinical studies and in further service evaluations of this programme. Primarily this relates to the nature of this being a service evaluation and not a form of designed controlled trial or audit. These data may provide hypotheses for future controlled evaluations of such programmes in acutely unwell SMI patients. There are no data on the number of patients who were available for the intervention but were either not approached or declined. Thus it cannot be certain that our results can be extrapolated to other inpatient populations. There is also no data on the severity of the patient's illness and there is no control group. All data obtained from this service evaluation has been included, however data on many demographics including pharmacological treatments, previous weight changes and BMI were not available for 38% of the patients and have thus not been reported. A final limitation is the short follow up period that fails to assess whether any benefits may persist.

## Conclusion

Despite these limitations there is an increasing body of evidence that weight management interventions may potentially provide benefit [12,21] although caveats remain that most studies are small, use varying interventions and are mostly short term. Further clinical studies are required in acutely psychotic patients to determine if early weight gain can be prevented or ameliorated and benefits maintained longer term. Soon after diagnosis of psychosis and soon after drug initiation in more chronic subjects, may represent crucial times to intervene with regard to prevention of weight gain.

## Competing interests

CB and DM are employees of Eli-Lilly UK.

Full details of the programme and its application can be obtained from via Email: – bushe_chris@lilly.com

This paper has been written by the named authors without editorial assistance.

## Authors' contributions

CB, DM, PD, MF and CH conceived the analysis. CB analysed the data. PD, MF and CH have expertise in the SFW for outpatients. CB drafted the manuscript and all authors had input and approved the final version.

## Pre-publication history

The pre-publication history for this paper can be accessed here:


